# Risk stratification and scoring systems in upper and lower gastrointestinal bleeding: review of performance and limitations in the emergency department

**DOI:** 10.3389/fmed.2025.1564015

**Published:** 2025-06-20

**Authors:** Fatemeh Mohammadyari, Amirreza Aflak, Mahta ZareDini, Reza Irandoost, Saeed Ghaffari, Arsham Gharib, Minaalsadat Roshanaei, Mazyar Tahavori, Sepehr Olangian-Tehrani

**Affiliations:** ^1^School of Medicine, Guilan University of Medical Sciences, Rasht, Iran; ^2^Student Research Committee, Tabriz University of Medical Sciences, Tabriz, Iran; ^3^Student Research Committee, Mashhad University of Medical Sciences, Mashhad, Iran; ^4^School of Medicine, Rafsanjan University of Medical Sciences, Rafsanjan, Iran; ^5^Department of Surgical Technology, Faculty of Medicine, Shiraz University of Medical Science, Shiraz, Iran; ^6^Islamic Azad University Pharmaceutical Sciences, Tehran, Iran; ^7^Gastrointestinal and Liver Diseases Research Center, Iran University of Medical Sciences, Tehran, Iran; ^8^School of Medicine, Iran University of Medical Sciences, Tehran, Iran; ^9^Avicennet, Tehran, Iran

**Keywords:** gastrointestinal hemorrhage, scoring method, prognosis, GIB, emergency, triage

## Abstract

**Background:**

Gastrointestinal bleeding (GIB), which includes both upper GIB (variceal and non-variceal) and lower GIB, represents a significant cause of emergency department referrals.

**Main body:**

Given the potential risks of blood transfusion, re-bleeding, and mortality in these patients, it is essential to establish a system for prioritizing critical patients. Several risk stratification scoring systems have been developed based on patients' clinical characteristics and/or endoscopic findings. However, the optimal scoring system for each clinical scenario remains uncertain.

**Conclusion:**

In this study, we design the first comprehensive review and compare almost all of the upper gastrointestinal bleeding (UGIB), as well as lower gastrointestinal bleeding (LGIB) risk stratification scoring systems individually regarding their advantages, disadvantages, and limitations.

## Graphical Abstract

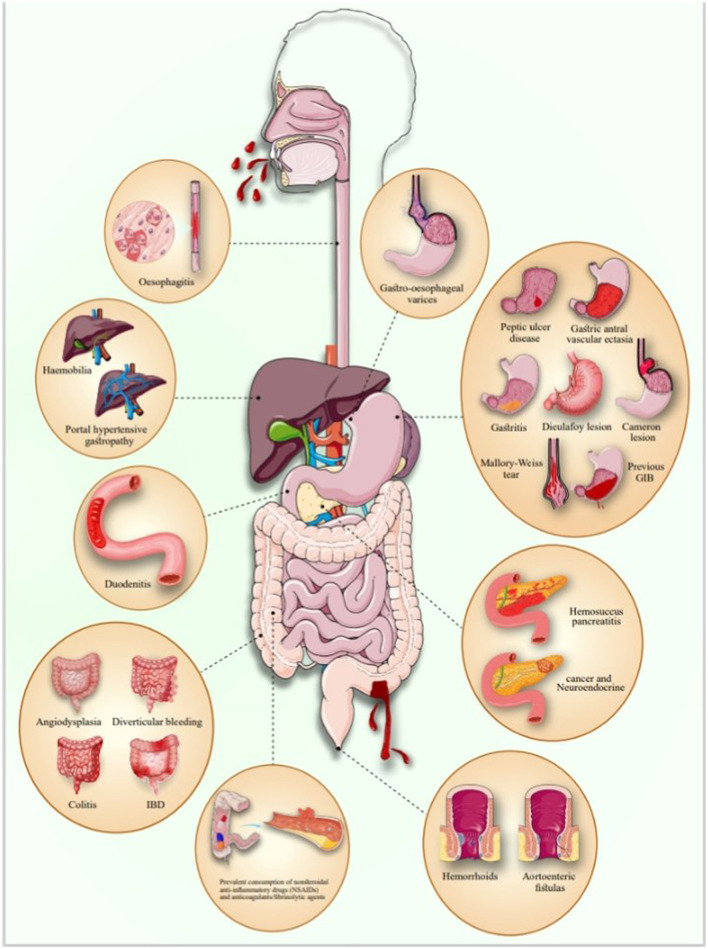

## 1 Introduction

GIB refers to any bleeding that originates in the gastrointestinal (GI) tract, which includes the esophagus, stomach, small intestine, large intestine, rectum, and anus. GIB based on the Treitz ligament is divided into UGIB and LGIB (1). GIB can result from a variety of underlying conditions affecting the entire digestive tract, from the esophagus to the anus. Common causes of UGIB include peptic ulcer disease (PUD), esophageal varices, Mallory-Weiss tears, tumors, angiodysplasia, and the use of medications such as non-steroidal anti-inflammatory drugs (NSAIDs) and anticoagulants. In contrast, LGIB may arise from hemorrhoids, diverticulitis, anal fissures, inflammatory bowel disease (IBD), colon polyps, and colorectal cancers. Identifying the root causes is crucial for effective management and treatment of GIB (2).

UGIB commonly presents either as hematemesis or melena whereas LGIB usually presents as hematochezia. The severity of GIB can differ from insignificant hemorrhage to extreme active bleeding. Most of the cases stop bleeding before reaching the emergency department (ED). Still, approximately 70–80 % of high-risk patients, such as those having a visible vessel, get into the ED with active bleeding and are at a higher risk for rebleeding and may require therapeutic interventions (3).

The assessment of these patients is challenging for physicians in the ED due to different factors such as different bleeding sites, severity of hemorrhage, and risk of rebleeding. Rapid diagnosis, efficient resuscitation, and risk management can improve outcomes and lower mortality and morbidity rates (4). Therefore, it is crucial to categorize patients into those who need hospitalization and immediate procedures and who can be discharged or treated electively. This will reduce the burden of hospital stays, medical expenses, and pressure on the health system (3, 5, 6). To reach this goal, prognostic scoring systems that consider clinical and/or endoscopic findings are used to evaluate the intervention, need for transfusion, intensive care unit (ICU) admission, rebleeding risk, admission duration, and risk of morbidity and mortality (7, 8).

The most studied risk stratification system for UGIB is the Glasgow Blatchford Score (GBS), which helps predict the necessity of blood transfusion and urgent endoscopy. However, it also has limitations, such as identifying patients who need monitored beds, due to overlooking risk factors and comorbidities such as alcohol withdrawal, age, aspiration pneumonia, and respiratory problems (9). In addition to GBS, there exists a variety of scoring systems, each designed to address a specific problem and each with its own strengths and weaknesses. Notable scoring systems include the Pre-Endoscopic Rockall score (pRS), AIMS65 score, and Modified Early Warning Score (MEWS), to name a few. Each scoring system has its specific applications, and their usage depends on the clinical scenario and the clinician's expertise.

This review evaluates current scoring systems for both UGIB and LGIB in emergency settings, comparing their strengths, weaknesses, and practical applications, while also exploring the potential of AI-driven models in the future of GIB risk prediction and management. By understanding these tools, clinicians can make better-informed decisions, leading to improved patient outcomes and more efficient use of healthcare resources.

## 2 UGIB

Risk stratification in UGIB has been the focus of extensive research, resulting in the development of several scoring systems tailored to predict outcomes such as mortality, re-bleeding, the need for intervention, and hospitalization.

### 2.1 UGIB-specific scoring systems

#### 2.1.1 Glasgow Blatchford Score (GBS)

GBS is a risk evaluation tool that categorizes patients with UGIB into high- or low-risk groups. The GBS was created in 2000 in the United Kingdom, utilizing data from 1,748 patients. It helps predict the necessity for interventions and clinical outcomes. The original purpose of the Glasgow-Blatchford risk stratification score is to forecast adverse clinical results in the general population of patients with UGIB who present to the ED (10).

The GBS does not depend on the results of endoscopic examinations. Instead, it is based on easily measurable clinical and laboratory variables. Given its non-reliance on endoscopic findings, the GBS offers a unique approach to risk assessment that is particularly valuable in emergency settings. The GBS incorporates clinical factors such as systolic blood pressure (SBP), pulse rate, the existence of hepatic disease, the existence of melaena, presentation with syncope, and heart failure, as well as serological parameters such as urea and hemoglobin (Hb) levels. These variables can be easily assessed during the initial evaluation, enabling clinicians to identify appropriate patients for outpatient management (11).

Furthermore, a GBS score of ≥2 could serve as a threshold for determining the need for hospitalization for UGIB (11). Additional research has demonstrated that the GBS is effective in predicting the likelihood of rebleeding and the necessity for transfusion in patients with UGIB (9). In one particular investigation, their findings suggest that patients with GBS scores >7 have an increased threat of significant bleeding that may require endoscopic or surgical intervention, as well as an increased threat of death (12). Another research indicated that patients at high risk with GBS scores exceeding 12 experience decreased mortality rates if they underwent endoscopy within 13 h of their initial presentation (13).

According to the evidence, patients with GBS scores over seven should have an endoscopy within 24 h of their presentation and potentially sooner for those with GBS scores over 12. Patients with GBS scores between 4 and 7 require only endoscopic therapy without significant adverse outcomes, indicating that this intermediate-risk category should have inpatient endoscopy, although not essentially within 24 h of presentation (14).

In addition, the GBS distinguishes itself from the RS by not taking patient age into account when assessing treatment needs, resulting in a more precise evaluation, especially for younger patients at risk of severe bleeding. Furthermore, the GBS demonstrates a stronger correlation with the length of hospital stays, highlighting its effectiveness in clinical environments. Its capability to predict both the necessity for interventions and the anticipated duration of hospitalization renders the GBS an essential resource for healthcare professionals treating patients with UGIB (10).

However, the GBS has several limitations that affect its practical usage in clinical settings. One important drawback is the complexity and time required for calculating clinical scores such as the GBS, which can deter their use in fast-paced environments where physicians may find that these scores do not substantially enhance their decision-making capabilities (15). In addition, clinicians are prevalently concerned about managing elderly patients as outpatients, even when their GBS score is 0. This apprehension underscores the demand for an age-modified version of the GBS, particularly when there are clinical issues about the risks associated with avoiding hospitalization for older patients (15). Furthermore, a study indicated that the GBS exhibited a lower discriminative value than earlier research findings. This discrepancy may be attributed to the study's narrower definition of intervention, which was confined to radiological or surgical procedures, whereas earlier studies encompassed a wider array of interventions (16). These limitations highlight the need for ongoing evaluation and potential modifications to the GBS to enhance its utility in diverse clinical scenarios.

#### 2.1.2 Modified Glasgow-Blatchford Score (mGBS)

The mGBS, which includes variables such as pulse rate, SBP, blood urea nitrogen (BUN), and Hb, is a simplified version of the original GBS that predicts the demand for interventions such as blood transfusion, endoscopic treatment, or surgery. It improves upon the GBS by eliminating anamnestic variables that may involve interpretation or subjective judgment, reducing the risk of bias. These variables, including syncope, hepatic disease, cardiac disease, and melena, are not included in the mGBS, resulting in a scoring range from 0 to 16. This simplification of the score makes it easier to adopt and more suitable for routine clinical practice (17).

In a retrospective study, the mGBS and the GBS were highly accurate in predicting the requirement for urgent intervention or death. Utilizing these scores can facilitate decision-making. Nevertheless, it is critical to note that clinical judgment remains crucial, particularly in patients with low-to-moderate risk (18).

In addition, compared to other scoring systems, the mGBS and the GBS appeared to be more effective in forecasting the requirement for medical interventions or the threat of death. Importantly, these scores were explicitly designed to check the need for interventions, while the PERS and AIMS65 scores were created to estimate mortality risk. From a clinical perspective, predicting the need for an intervention is more pertinent than predicting in-hospital mortality as the main goal of risk stratification is to identify patients who can safely be discharged from the ED (18).

Consequently, the mGBS is a valuable tool for predicting the demand for therapeutic interventions, comprising endoscopy, in scenarios of UGIB (17, 19). It is worth noting that there is limited research available on this topic (17, 19, 20).

#### 2.1.3 Glasgow-Blatchford Score combined with nasogastric aspirate

Nasogastric tube placement and estimation of aspirate have been inquired as a diagnostic tool to assess the hazard in patients suspected of having UGIB. This procedure is advantageous due to its availability, low cost, and minimal threat of complications (21, 22).

Several studies have found that the presence of a bloody nasogastric aspirate is related to active bleeding in patients with UGIB (23–25). In addition, bloody nasogastric aspirate is to be correlated with high-risk endoscopic lesions (HRELs) detected during endoscopy and a higher chance of bleeding again (26–29).

A novel procedure that combines the GBS and nasogastric aspirate (NGA) has been shown to enhance the diagnostic precision of the GBS. This process not only improves the prediction of patients without HRELs but also outperforms the GBS alone in this regard (30).

A study on nasogastric lavage for predicting non-variceal UGIB (NVUGIB) found that its value varies based on the GBS. Adding nasogastric lavage findings to the GBS improved the prediction paradigm for patients with GBS ≤ 11 but was not beneficial for those with GBS ≥ 12. This suggests that nasogastric lavage can provide additional information for UGIB in patients with GBS ≤ 11 but is not helpful for high-risk patients with a GBS of 12 or higher, for whom nasogastric lavage should not be postponed until after endoscopic examination (31).

#### 2.1.4 Rockall score (RS)

According to the Rockall scoring system, there are five independent risk factors associated with mortality following UGIB: age, shock, comorbidity, diagnosis, and major stigma observed during endoscopy. In addition, there is a pre-endoscopic Rockall score (PERS) which is calculated independently of the endoscopic findings. The complete Rockall score ranges from 0 to 11, while the pre-endoscopic Rockall score ranges from 0 to 7. Higher scores indicate higher risk of mortality, re-bleeding, and prolonged hospital stays (32–36). However, there are controversies regarding the optimal cutoff points for categorizing patients into low- and high-risk groups (35, 37–41).

In Johnson et al. (42) study, almost one-third of patients with a PERS of 0 required intervention, such as blood transfusion and/or endoscopy. The analysis demonstrated a significant correlation between PERS and 30-day mortality with 45% increased risk for each one-point increase in PERS. However, another study reported an association between RS >3 and re-bleeding, surgery, and death (41).

Garcia et al. (43) identified RS cutoff points with the highest sensitivity and specificity for predicting mortality and re-bleeding within 30 days of UGIB, which were 5 and 6, respectively. The area under the ROC curve (AUC) was 0.76 (CI: 0.68–0.84) for mortality risk, 0.71 (CI: 0.55–0.88) for re-bleeding risk, and 0.66 (CI: 0.58–0.74) for the need for more than two units of blood transfusion. In contrast, Lip et al. (44) concluded low discriminative ability of the RS for these outcomes in NVUGIB. However, patients with RS scores ≥ 8 were considered as a high-risk group and showed higher rates of mortality and re-bleeding.

In a prospective cohort study, Frias et al. (38) assessed the utility of pre- and post-RS in predicting overall mortality, UGIB-related mortality, and re-bleeding in NVUGIB patients at admission, as well as at 1- and 3-month follow-ups. The complete RS showed a high discriminatory ability for UGIB-related mortality at all-time points, as well as for re-bleeding at the 1- and 3- months follow-ups. A complete RS cutoff for safe discharge of patients with 100 sensitivities was <6 point. Moreover, an RS score ≥ 3 was deemed a reliable threshold for determining the need for further follow-up (38).

Building on the notion that combining endoscopic findings with clinical parameters improves prognostic prediction in UGIB, several studies confirm the superiority of the complete RS over the PERS (18, 38, 45, 46). A longitudinal cohort study demonstrated a significantly higher risk of re-bleeding in patients with an initial RS of ≥ 6 after a follow-up period of 3.5 years (47). Similarly, a retrospective study showed significantly higher rates of re-bleeding and mortality over a 3-year period in patients with a Rockall score of 4, compared to those with scores of 0 and 1 (48).

Although it is generally expected that endoscopic findings in combination with clinical parameters provides better prognostic prediction of UGIB outcomes, some inconsistencies remain regarding the superiority of the complete RS over the PERS (46, 49), which requires more prospective studies with larger sample size to resolve these discrepancies.

In several studies, the pre- and post-endoscopy RS have been compared with the Glasgow-Blatchford Score (GBS) and other scoring systems (18, 45, 46, 49–51).

The GBS and its modified versions demonstrated the highest discriminatory capacity for predicting in-hospital death or the need for intervention with AUC values of 0.77 (95% CI: 0.75–0.80) and 0.78 (95% CI: 0.76–0.81), respectively (18). Similarly, when compared with RS and PERS, GBS showed superior predictive power in terms of mortality (AUC: 0.901 for GBS vs. 0.735 for RS, with a cutoff point of 4.5, and 0.844 for PERS, with a cutoff point of 3.5) and the need for transfusion. In contrast, RS had a higher AUC for re-bleeding (0.981) (cutoff point =7.5) vs. 0.759 for GBS vs. 0.885 for PERS (cutoff point =4.5) (46).

According to Morarasu et al. study (51), GBS was found to be a superior predictor of surgical interventions, although it showed unsatisfactory discrimination in another study (45).

Custovic et al. (49) and Lu et al. (45) conducted the same analyses, finding that RS and PERS were superior to GBS in predicting mortality.

An RS cutoff of >3.5 was identified as the best threshold for predicting the need for endoscopic treatment, with a sensitivity of 77.8% and specificity of 50% (49).

Despite ongoing debate over the ability of different scoring systems to predict mortality and the need for intervention, the AUC for re-bleeding is higher for PERS and RS, while GBS is more effective in predicting the need for blood transfusion (18, 45, 46, 49).

#### 2.1.5 AIMS65 score

In 2011, Saltzman et al. (52) developed a new simplified risk stratification scoring system for UGIB, known as AIMS65, based on five readily available factors in the emergency department (ED): albumin, international normalized ratio (INR), mental status, systolic blood pressure (SBP), and age. The scoring system demonstrated high discriminatory capacity for mortality (AUC = 0.80), similar to the findings from a validation study (AUC = 0.77). Several studies have established AIMS65 scores of <3 or <2 as a cutoff points for identifying patient with low-risk in-hospital mortality (53–56). AIMS65 scores > 3 were associated with 100% in-hospital mortality (57). Higher scores were associated with longer hospital stays and increased costs (*p* < 0.001) (52). Among patients with peptic ulcers, scores >3 were correlated with a greater need for pre- and post-endoscopic blood transfusions (58). Nevertheless, the AIMS65 was unsatisfactory for predicting transfusions and 30-day re-bleeding (AUC = 0.612, and 0.529, respectively) (59).

The GBS, RS, and AIMS65 are widely applied risk stratification tools in UGIB, each with distinct clinical applications. GBS is a pre-endoscopic score that emphasizes clinical and laboratory parameters, excelling in early triage and predicting the necessity for intervention, particularly transfusion (9, 11). RS incorporates both clinical and endoscopic findings and is superior in anticipating rebleeding and mortality post-endoscopy, although its dependence on endoscopic data limits early utility (32, 38, 41, 46). AIMS65, composed of five simple pre-endoscopic variables, provides rapid and accurate forecast of in-hospital mortality but performs poorly in identifying rebleeding risk or requirement for intervention (53, 55, 57, 59). While GBS offers high sensitivity for urgent care decisions, RS facilitates comprehensive risk stratification, and AIMS65 allows for efficient mortality evaluation at admission. Notably, GBS excludes age, limiting its mortality prediction (12, 15), whereas AIMS65 includes age and other systemic indicators. These tools, therefore, cater complementary roles in optimizing UGIB management at different phases of clinical care.

### 2.2 Other UGIB scoring systems

#### 2.2.1 NVUGIB models

The Progetto Nazionale Emorragia Digestiva (PNED) score by Marmo et al. (60) is a reliable prognostic tool for predicting mortality in patients with NVUGIB. Population was 1,360 cases, and AUC is 0.81. It was developed as an alternative to existing scoring systems such as the RS to provide a more accurate assessment of mortality risk in these patients. The PNED score is calculated by age, Hb, SBP, BUN, and comorbidities (60).

The CHAMPS score by Matsuhashi et al. (61) is a prediction score that was made to assess NVUGIB mortality. Population was 2,200, and c statistic is 0.91. This score is made up of six components: comorbidities, HB, age, malignancy, pulse rate, and shock (61).

#### 2.2.2 Acute UGIB (AUGIB) models

The LHS by Maks et al. (3) predicts intervention for AUGIB patients. Population was 404, and AUROC is 0.82. This score uses serum Hb and creatinine (Cr), HR, and chronic liver disease as positive factors and C-reactive protein (CRP) and alternative diagnosis as negative factors (3).

Cologne-WATCH (C-WATCH) score, a pre-endoscopic risk predictor by Hoffmann et al. (62), can be used for patients with AUGIB including variceal hemorrhage. Population was 908, and AUC is 0.704. C-WATCH is calculated by CRP, WBC, alanine aminotransferase (ALT), thrombocytes, Cr, and Hb (62).

Canada-United Kingdom-Adelaide (CANUKA) score by Oakland et al. (63) predicts the risk of AUGIB. Population was 10,639, and AUROC is 0.77. The 10 used factors are age, melena, hematemesis, syncope, liver disease or malignancy, HR, SBP, Hb, and serum urea level (63).

#### 2.2.3 Other models

The T-score by Tammaro et al. (64) predicts the need for urgent endoscopic intervention in UGIB patients. Population was 602, and ROC curve is 0.72. T-score uses Hb, SBP, pulse rate, and general conditions. This score determines when to do esophagogastroduodenoscopy (OGD) for UGIB cases (64).

H3B2 by Sasaki et al. (65) helps find urgent UGIB. Population was 675, and AUROC is 0.73. H3B2 includes three factors starting with H (hematemesis, HR, and Hb) and two factors starting with B (BP and BUN) (65).

The MAP (ASH) score by Redondo-Cerezo et al. (66), a straightforward pre-endoscopy assessment tool, exhibits notable efficacy in forecasting the necessity for intervention and mortality in UGIB cases. Population was 3,012, intervention AUROC is 0.83, and mortality AUROC is 0.74. This score consists of six components: GCS, ASA score, pulse rate, ALB, SBP, and Hb (66).

The Rebleeding-Comorbidities-Deteriorating (Re.Co.De) score by Marmo et al. (67) predicts the mortality of UGIB cases based on 10 components: altered mental status, American Society of Anesthesiologists (ASA) score, sepsis, renal and respiratory failure, ascites, acute myocardial infarction (MI), re-bleeding, multi-organ dysfunction, and disseminated malignancy. Population was 2,764, and AUROC is 0.94. Re.Co.De score separates 1-month mortality rate in three classes by number of present factors (67).

Hirosaki or lino score, also known as Japanese score, by Lino et al. (68) predicts need for endoscopy for UGIB cases. Population was 212, and AUROC is 0.85. Hirosaki score has SBP, syncope, Hb, hematemesis, and BUN as positive factors and antiplatelet agents and estimated glomerular filtration rate (eGFR) as negative factors (68).

Baylor bleeding score (BBS) by Saeed et al. (69) is a score for bleeding PUDs based on pre-endoscopic factors and post-endoscopic factors. Population was 47. Pre-endoscopic factors are age, severity, and duration of related diseases, and post-endoscopic factors are the position and type of bleeding (69).

Modified Nagoya University score (modified N-score) by Ito et al. (70) is an endoscopy predictor specific for patients with melena. Population was 721, and AUROC is 0.731. Modified N-score has syncope, BUN level, and BUN/Cr ratio as positive factors and anticoagulant drug use as negative factor (70).

Table 1 shows strengths and limitations of these UGIB scores.

**Table 1:** A summary of the strengths and limitations of UGIB scoring systems.

**Score name**	**Strength**	**Limitation**	**References**
**UGIB scoring systems**			
GBS	Accurate for triage and transfusion prediction	Limited mortality prediction; excludes age	(9, 11, 15)
Rockall score	Strong mortality and rebleeding prediction (post-endoscopy)(38, 41)	Requires endoscopy; variable cut-off thresholds (34, 37, 38, 41)	(34, 37, 38, 41)
AIMS65	Simple; Strong predictor of mortality in both variceal & non-variceal UGIB (51)	Poor for rebleeding/intervention; underperforms in predicting transfusion (59)	(51, 59)
PNED score	Better than GBS and RS and similar to AIMS65 in predicting mortality		(60)
CHAMPS score	Calculated by a free application better than GBS, AIMS65, ABC score and RS in predicting mortality		(61)
LHS	Better than GNS in predicting intervention need		(3)
C-WATCH score	Similar to RS, p-RS, and GBS in predicting mortality		(62)
CANUKA score	Multicentered data from 3 countries better than GBS in predicting mortality	Weaker than GBS in predicting RBC transfusion and endoscopic therapy	(63)
T-score	Better than ABC, AIMS65, and pRS in predicting intervention need		(64)
H3B2 score	Better than GBS, AIMS65, MAP score, and hirosaki score in predicting hemostatic treatment better than GBS, MAP score, and hirosaki score in predicting mortality	Weaker than the AIMS65 in predicting mortality	(65)
MAP (ASH) score	Better performance in older UGIB cases similar to GBS in predicting intervention need similar to AIMS65 in predicting rebleeding		(66)
Re.Co.De score	Better than ABC, RS, GBS, PNED, and AIMS65 in predicting mortality		(67)
Hirosaki/lino score	Better than GBS, CRS, ABC, AIMS65, and MAP scores in predicting endoscopy need	Weaker than GBS in higher levels of hemoglobin	(68)
BBS		Weaker than GBS in predicting intervention, re-bleeding and transfusion weaker than RS in predicting lethal outcomes	(69)
modified N-score	Specific for patients with melena easy to calculate better than GBS in predicting endoscopy need		(70)
**General scoring systems**			
MEWS	Simple calculations High specificity for predicting follow-up bleeding (94.1%). Better results compared to other scoring systems in forecasting in-hospital mortality for patients aged 65 and 75 years and older (AUC: 0.82).	Studies are single-centered, with short follow-up periods, and overlooking coexisting conditions that affect outcomes. A low predictive capability for assessing the need for endoscopic therapy has been observed, with AUC of 0.51. Reports indicate inconsistent performance metrics across various populations (Asian and non-Asian) and clinical settings.	(102–107)
SOFA score	Better than AIMS65, RS, GBS, and ABC in predicting mortality in ICU	Weaker than APACHE II in predicting mortality in ICU not specific for GIB	(108)
qSOFA score	Easy to calculate better than GBS and RS in predicting ICU need	Weaker than GBS in predicting lethal outcomes and transfusion need	(109)
APACHE II	Better than SOFA score, GBS, AIMS65, RS, and ABC in predicting mortality	Weaker than AIMS65 and SOFA score in predicting length of hospitalization not specific for GIB	(110)
CCI	Better than GBS in predicting mortality and re-bleeding risk	Not specific for GIB	(111)
PI	Noninvasive and easy useful in crowded EDs higher sensitivity than the RS in predicting mortality	Lower specifity than the RS in predicting mortality not specific for GIB	(112)
**Novel scoring systems**			
ML score by Deshmukh et al.	Better than APACHE IVa score in predicting mortality	Lack of comparison with other GIB scores	(113)
ML score by Herrin et al.		Not appropriate for acute or subacute cases lack of comparison with other GIB scores	(114)
ML score by Ungureanu et al.	Combination of GBS, RS, BBS, AIM65, and T-score in predicting mortality lesser number of parameters than other ML models for GIB (33)		(115)
ML score by Veisman et al.	Better than GBS and RS in predicting intervention need		(116)
LSTM by Shung et al.		Lack of comparison with other GIB scores	(117)
BLOVO infant score	First endoscopy score specific for infants	Lack of comparison with other GIB scores	(118)
CTP	Good prognostic value for predicting mortality in AVB patients or cirrhotic patients with AVB.	Subjective variables (ascites and hepatic encephalopathy). Need for imaging. Containing INR variable which is not reliable for liver function in cirrhotic patients.	(7, 121, 124, 127, 130)
MELD	Good prognostic value for predicting mortality in AVB patients or cirrhotic patients with AVB. Containing objective variable compare to CTP.	Complex calculation Containing INR variable which is not reliable for liver function in cirrhotic patients.	(7, 121, 124, 127, 130)
PALBI score	Better than MELD score in both predicting mortality and re-bleeding	Mathematical calculation including logarithm weaker than AIMS65 and GBS in predicting mortality	(132)
HOPE-EVL	Ease of calculating accurate prognostic value for in-hospital mortality following EVL in esophageal variceal bleeding.	This score is only examined in Japan. This score only applies for patients underwent EVL (cannot apply for those treated with other ways like pharmacological therapies).	(130)

## 3 LGIB

LGIB is characterized by any form of bleeding originating from the lower gastrointestinal tract, i.e., distal to the ligament of Treitz (71).

LGIB tends to be a self-limiting condition. Nonetheless, a subset of patients with LGIB may encounter adverse events. Therefore, it is crucial to conduct risk stratification for these patients to enhance the selection process for emergency hospitalizations and ensure safe discharge (72, 73).

This section provides a concise overview of the most studied risk scores in this category, highlighting their respective limitations and advantages in comparison with one another.

### 3.1 LGIB-specific scoring systems

#### 3.1.1 Oakland score

To create a risk score that could effectively direct discharge decision-making, Oakland et al. (74) evaluated 2,338 patients with LGIB from 143 institutions in the UK.

The score has eight simple variables that allow pre-endoscopic evaluation of patients in Emergency Departments including age ≥40, male sex, history of previous acute LGIB admission, heart rates ≥70, bloody digital rectal examination (DRE) findings, and finally, low hemoglobin (Hb) and systolic blood pressure (SBP) levels as the most significant risk factors for adverse outcomes.

Compared to existing UGIB and LGIB scores such as AIM65 (AUC:0·62), Rockall (AUC:0·64) BLEED (AUC, 0·63), NOBLADs (AUC, 0·65), and Strate (AUROC, 0·69) scores, Oakland (AUC, 0·84) and Blatchford's (AUC, 0·80) scores performed better in predicting safe discharge (5, 74). Moreover, it sustained its predictive accuracy within the cohort of emergency department patients (75).

However, although the original cutoff score of 8 points or lower demonstrates a high sensitivity (98.4%) on identifying high-risk patients, its specificity (16%) to recognize low-risk patients that can be safely discharged is limited. Therefore, several investigators, including the original authors, suggested expanding the cutoff score for safe discharge to address this issue (76, 77).

Second, regarding the substantial weighting of this score on hemoglobin levels, the presence of baseline anemia in the population may restrict the score's performance (78).

Nevertheless, when it comes to forecasting safe discharge, risk scores that include Hb levels as one of their components—such as GBS and Oakland scores—generally perform better than other risk scores (79, 80). In all, Oakland score performs better than GBS among patients with LGIB, and when the origin of bleeding cannot be precisely determined, GBS is favored over the Oakland score to guide decision-making (74, 81). On the prediction of 30-day mortality among LGIB patients, however, studies demonstrated that ABC (AUC, 0.84) score outperformed Oakland (AUC, 0.69) and GBS (AUC, 0.74) scores (82, 83).

#### 3.1.2 SHA(2)PE score

The SHA(2)PE score was devised and validated by Hreinsson et al. in Iceland in 2018 by utilizing data from 6,646 upper endoscopies and 6,346 colonoscopies.

The score's main goal was to determine which patients would be best suited for outpatient care.

Regarding this, they developed another weighted risk score, which is comparable to the Oakland score, that identifies low Hb levels as the primary risk factor for adverse outcomes and SBP <100 mmHg, history of anti-platelet therapy, anticoagulant therapy, heart rates (HR) > 100 bpm, and emergency room (ER) bleeding as other prognostic factors (84).

Subsequent studies indicated that the SHA(2)PE (AUC, 0.797) demonstrated inferior efficacy compared to the Oakland score (AUC, 0.85) in recognizing high-risk patients for safe discharge (85, 86).

On the other hand, when it came to forecasting prolonged hospital stay, SHA(2)PE performed better (83).

Conversely, in predicting prolonged hospitalizations lasting 10 days or more, SHA(2)PE score demonstrated superior performance (AUC: 0.721) compared to Oakland, GBS, AIMS65, ABC, ROCKALL, and CHAMPS scores (87).

#### 3.1.3 NOBLADS score

The NOBLADS score was developed and validated in 2016 by Naoki et al. in Japan by analyzing 439 patients' data to predict severe LGIB.

NOBLADS score consists of eight factors, each of which adds one point to the final result: NSAIDs or antiplatelet consumption, lack of diarrhea or abdominal tenderness, SBP ≤ 100 mmHg, syncope, albumin levels <3.0 g/dL, and disease scores of ≥ 2.

Unlike Oakland score, Hb levels were not a component of this score. Nevertheless, it introduced non-aspirin antiplatelet use, no diarrhea, and low albumin levels as novel risk factors for the severity of LGIB (88).

NOBLADS score performed better than earlier scores (BLEED, Strate, Velayos, and Newman AUROC: 0.61, 0.71, 0.72, and 0.69, respectively) in predicting the severity of LGIB owing to the NOBLADs (AUROC, 0.77), score's novel risk factors (88–90). However, this score did not outperform Oakland score in predicting major bleeding events (AUROC, 0.58 vs. 0.93) (5).

#### 3.1.4 Strate score

In 2003, Strate et al. evaluated 252 patients who were hospitalized with ALGIB and identified seven risk factors for severe LGIB (91).

The components of the score are SBP ≤ 115 mmHg, HR ≥ 100 bpm, a history of syncope, bleeding noticed during the initial 4 h of evaluation, a non-tender abdominal examination, the use of aspirin, and a disease score ≥ 2 using the Charlson comorbidity scale (92).

This risk score has the advantage of not requiring blood tests.

However, according to a study by Xavier et al., no correlation was found between the Strate score and adverse events associated with LGIB, and barely 1% of patients were assigned to the low-risk group that could be safely discharged (93).

Moreover, this score did not predict LGIB outcomes any better than the Oakland score, apart from the requirement for hemostasis (AUROC, 0.82 vs. 0.36) (5).

#### 3.1.5 CACHEXIA score

Tominaga et al. developed and validated this recently introduced score in Japan by studying 8254 LGIB patients who initially fulfilled the criteria for ED admission, aimed to predict 30-day mortality and 1-year mortality among LGIB patients with precision.

The CACHEXIA score is an acronym representing various clinical parameters: Cancer (including metastasis tumor, blood tumor, and bleeding from tumor), Albumin levels, Cirrhosis, High Performance Status (PS), Extremely thin (i.e., low body mass index), Increased levels of C-reactive protein (CRP) and Blood Urea Nitrogen (BUN), and Anemia (i.e., requiring blood transfusion).

In addition, six components of the long-term scale serve as predictive indicators for the short-term mortality scale.

The CACHEXIA score (AUC, 0.90, for validation) exhibited superior performance compared to the NOBLADS, Oakland, and Sengupta et al. (AUC: 0.84, 0.71, and 0.89, respectively) scores regarding short-term mortality, while also demonstrating a good performance (AUC: 0.84, for validation) in predicting long-term mortality (94).

Nevertheless, further research is required to externally validate these findings across diverse populations, especially among LGIB patients who could be safely discharged initially.

In Table 2, the strengths and limitation of these mentioned scores are summarized.

**Table 2:** A summary of strengths and limitations of LGIB scoring systems.

**Score name**	**Strengths**	**Limitations**	**References**
Oakland score	Outperformed other LGIB scores in predicting safe discharge	Baseline anemia in the population may restrict the score's performance	(3, 5, 76–78)
	High sensitivity	Low specificity	
SHA(2)PE score	Outperformed Oakland score in predicting prolonged hospital stay	Inferior performance compared to the Oakland score in recognizing high-risk patients for safe discharge	(83, 85, 86)
NOBLADS score	Outperformed earlier scores (BLEED, Strate, Velayos, Newman) in predicting the severity of LGIB	Did not outperform Oakland score in predicting major bleeding events	(5, 88–90)
STRATE SCORE	Not requiring blood work	Poor performance in subsequent researc	(5, 92, 93)
	Outperformed Oakland score in predicting need for hemostasis	Did not predict other LGIB outcomes any better than the Oakland score	
CACHEXIA score	Outperformed NOBLADS, Oakland, and Sengupta scores in predicting short-term mortalit	Requires external validation	(94)
	Good performance in predicting long-term mortality		
BLEED score	Aimed to predict the adverse outcomes in GIB	Poor performance in subsequent research	(93, 95, 100)
LONG-HOSP	Outperforms the existing LGIB scores in predicting prolonged hospital stays (16)	Complexity due to extensive number of components	(97)
		Requires external validation	
Sengupta et al.	Predicting 30-day mortality in patients with LGIB	Requires further reseach to externally validate and compare the score to existing LGIB scores.	(98)
ANN model	Aimed to predict mortality during hospital stay, re-bleeding, and necessity for therapeutic intervention	Time consuming and requirement of appropriate facilities	(99)
		Requires external validation	
Newman et al.	Aimed to predict the severity and adverse outcomes of LGIB	Requires external validation	(100)
Velayos et al.	Aimed to predict the adverse outcomes of LGIB	Requires external validation	(101)

### 3.2 Other LGIB risk scores

#### 3.2.1 Bleed score

The bleed score was developed and validated based on 108 ED admissions data to predict adverse outcomes in gastrointestinal bleeding. The high-risk group based on this score is defined as the presence of any of the five risk factors (ongoing bleeding from upper or lower GI sources on admission, SBP below 100 mm Hg, prothrombin time (Pt) above 1.2, unstable comorbid disease that necessitates ICU admission, and erratic or altered mental status by any primary or secondary cause) (95). The validation study indicates that patients with LGIB categorized in the high-risk group exhibited a higher rate of experiencing adverse outcomes compared to those in the low-risk group (96).

#### 3.2.2 LONG-HOSP score

The LONG-HOSP score was derived and validated in 2023 by analyzing 8,547 admission records and was specifically designed to predict prolonged hospital stays. The majority of this risk score's components are also present in other LGIB risk scores. However, for the first time, they also found that high BUN levels, diarrhea, abdominal pain, and tarry stool were significant risk factors for prolonged hospital stays (AUROC, 0.66, for validation) (97).

#### 3.2.3 Score by Sengupta et al.

Sengupta et al. developed and validated a risk scale in 2017 by employing pre-endoscopic data and machine learning methodologies within 6,104 LGIB patients, aiming to predict 30-day mortality in patients with LGIB. The components of this risk score can be categorized as either risk factors or protective factors, and they include the following: Age (ages under 40 serve as protective factors), dementia, chronic kidney disease (CKD), disseminated malignancy, use of systemic anticoagulants, chronic pulmonary disease, admission hematocrit, and albumin levels are identified as protective factors at elevated levels and risk factors at diminished levels. Patients with a low 30-day mortality risk were effectively identified by the risk score (AUC, 0.75, for validation) (98).

#### 3.2.4 ANN model

To evaluate the risk of mortality during hospital stay, re-bleeding, and necessity for therapeutic intervention in patients experiencing LGIB, DAS et al. created and validated separate artificial neural network (ANN) models patients tailored to each specific outcome by evaluating 190 LGIB. The models exhibited acceptable predictive capabilities for all three outcomes (AUC: 0.92, 0.93, and 0.95, respectively) (99).

#### 3.2.5 Score by Newman et al.

Newman et al. (100) conducted a study of 184 cases of LGIB to devise a risk score for predicting the severity and adverse outcomes of the condition. The risk factors correlating with severe bleeding were hematocrit <35%, bright red rectal bleed, and age>60 years. For the adverse outcome, the five variables sorted by their relative risk were creatinine > 150 μM, age> 60 years, abnormal hemodynamic parameters, continuous bleeding for first 24 h, and smoking (as a protective factor). The scores associated with these variables demonstrated discriminative capability (AUC, 0.79) (100).

#### 3.2.6 Score by Velayos et al.

In 2004, Velayos et al. studied a cohort of 94 patients presented with LGIB and proposed three main risk factors that may predict the adverse outcomes: a hematocrit level of 35% or lower upon admission, the presence of gross blood on DRE, and SBP <100 mm/Hg or a HR > 100 bpm 1 h after the initial evaluation (101).

Additional details regarding these scoring systems, including their strengths and limitations, are available in the Table 2.

## 4 General scoring systems

Several general scoring tools have been developed for early detection of clinical deterioration or prediction of mortality across a wide range of medical conditions, including GIB. These scores are not specific to gastrointestinal bleeding but can offer valuable insight, particularly in the ED setting.

### 4.1 MEWS

Subbe et al. (102) established the MEWS, first predicting disastrous medical situations such as ICU admission and cardiac arrest because of easy calculation and repetitiveness. MEWS comprises five key parameters: SBP, heart rate (HR), respiratory rate (RR), body temperature (BT), and the AVPU score (Alert, responds to Voice, responds to Pain, Unresponsive). In their study, 673 patients were evaluated. A score of 5 or more was associated with an increased risk of death, yielding an OR of 4.5 [95% CI: 2.8–10.7]. This study has demonstrated that raised MEWs scores are associated with increased mortality in a group of medical emergency admissions. It was determined that this score can predict unstable patients with any disease before the deterioration of their condition and can be a guide to prioritize patients for further interventions in ED (103–106).

Bozkurt et al. (106) showed that a MEWS score >4 could predict follow-up bleeding with a high specificity, but its ability was not proven statistically (p: 1.000). A total of 202 patients were included in the study. MEWS score was significantly correlated with hospital outcomes (p: <0.001). In addition, the non-surviving patients had a significantly greater median MEWS score compared to the discharged and hospitalized patients (p: 0.001 and p: 0.003, respectively). ROC curve analyses revealed a need of endoscopic intervention for MEWS score of >1. It also revealed that a MEWS score of >1 could convey the need for blood transfusion (p: 0.0470). A score of >2 significantly predicted death (*p* < 0.001).

In another study, Lai et al. (104) evaluated the scores of AIMS65, MEWS, GBS, CRS, and qSOFA on 442 consecutive cirrhotic patients. The researchers could not find significant differences in rebleeding prediction between AIMS65, MEWS, GBS, and CRS scoring systems (104). In addition, an AUC of 0.58 [95% CI: 0.53–0.64] was obtained for rebleeding prediction of MEWS.

Wu et al. (103) performed a study that compared six different predictive scores (pre-endoscopy RS, shock index (SI), age shock index (age SI), Rapid Acute Physiology Score (RAPS), Rapid Emergency Medicine Score (REMS), and MEWS). A total of 336 patients were recruited for the study, of whom 40 died. Their findings demonstrated that MEWS outperformed the other scores in predicting in-hospital mortality for patients over 65 years old (AUC: 0.82). In addition, MEWS was identified as the most effective tool for predicting in-hospital mortality among patients older than 75 years (AUC: 0.82) (103).

While the MEWS is a valuable tool, it should not supplant clinical judgment. It is crucial to consider the broader clinical context and various factors that may impact the patient's condition. Furthermore, the accuracy of MEWS can differ depending on the population being assessed and the specific clinical environment. Research has indicated that the performance of MEWS can vary significantly across different demographics and settings. Notably, studies conducted in non-Asian populations have reported higher predictive values compared to those observed in Asian populations, raising concerns regarding the general applicability of MEWS across diverse patient groups (107).

### 4.2 Sequential organ failure assessment (SOFA) score

This score by Vincent et al. (108) describes the degree of organ failure and the complications of critical illnesses. The SOFA score is not specific for UGIB, but it can be used for critical UGIB patients and predict their mortality. This score includes parameters of six organ systems: partial pressure of oxygen (PaO_2_), Glasgow Coma Scale (GCS), cardiovascular factors (mean arterial pressure (MAP), dopamine, dobutamine, and epinephrine/norepinephrine), BR, thrombocytes, and serum Cr. The efficacy of the SOFA score is confirmed by the European Medicines Agency (108).

### 4.3 Quick SOFA (qSOFA) score

The qSOFA score by Singer et al. (109) can identify UGIB patients with possible sepsis. This score uses RR, GCS, and SBP as the only three factors that make it easy to calculate (109).

### 4.4 Acute physiology and chronic health evaluation (APACHE) II

APACHE II by Knaus et al. (110) is a system for defining disease severity by temperature, MAP, HR, RR, PaO_2_, arterial pH, serum sodium, potassium and Cr, hematocrit (HCT), and white blood cells (WBCs). Like the SOFA score, APACHE II is not specific for UGIB, but it can be used for defining severity of critical UGIB patients (110).

APACHE Iva is a recent version of the APACHE score. This score has extra factors in comparison with APACHE II, including mechanical ventilation, GCS, thrombolysis, ICU administration information, and chronic diseases. Calculation of APACHE II is easier due to lower number of parameters.

### 4.5 Charlson Comorbidity Index (CCI)

This score by Charlson et al. (111) predicts mortality and comorbidity. CCI is not specifically a score for GIB, but it includes factors that affect GIB including liver diseases, chronic kidney diseases (DKD), and malignancies (111).

### 4.6 Perfusion Index (PI)

PI by Firat et al. (112) shows peripheral perfusion status non-invasively and easily by measuring pulsatile arterial flow. The population was 219, and AUC is 0.772. PI is used in several medical situations. PI can detect patients with UGIB early and can be very useful in crowded EDs (112).

Table 1 has discussed about strengths and limitations of general scoring systems.

## 5 Novel tools and non-traditional scoring systems

Emerging tools such as artificial intelligence (AI) and machine learning (ML)-based models have opened new pathways for personalized risk prediction in GIB. These systems can integrate large datasets and dynamic parameters to enhance decision-making in real time.

### 5.1 AI models

There is a growing utilization of ML-based models in evaluating and addressing acute GIB with the aim of prognostication and enhancing patient care. These models harness sophisticated algorithms and electronic health records to augment predictive capacities, guide triage decisions, and refine patient management strategies within ED contexts. The integration of artificial intelligence into healthcare frameworks holds the potential for reshaping the management approach for GIB patients in emergency settings.

An ML model was designed to predict mortality for GI bleeding by Deshmukh et al. (113). The population was 5,691, and AUC is 0.85. This model uses age, vital signs, bleeding place, GCS, intubation, readmission, antibiotics, antiarrhythmic, vasopressors, transfusion, metastatic cancer, immunosuppression, and laboratory factors including serum Cr, BUN, liver function tests, lactate, Hb, thrombocytes, coagulation tests, WBC, potassium, and serum bicarbonate as input; addition of endoscopic findings to these factors could improve predictive outcomes (113).

Few GIB predictors use antithrombotic treatment as a factor, but ML can solve this limitation. An ML score by Herrin at al. (114) has age, sex, ethnicity, cardiovascular diseases, antiarrhythmic drugs, gastroprotective agents, anticoagulants, antihypertensive drugs, NSAIDs, antiplatelets, selective serotonin reuptake inhibitors, antihyperlipidemic drugs, and baseline comorbidities as input. The population was 306 463, and AUC is 0.67. This model predicts GIB risk at months, so it is not appropriate for acute or subacute cases (114).

An ML model by Ungureanu et al. (115) predicts the mortality of NVUGIB patients. Population was 1,096, and AUC is 0.99. This model is a combination of GBS, RS, BBS, AIM65, and T-score and predicts mortality more accurately than scores mentioned before. Using this model is easier than other ML models due to lesser number of parameters (115).

An ML model by Veisman et al. (116) predicts endoscopic intervention for AUGIB patients. Population was 883, and AUC is 0.68. This model uses age, sex, pre-endoscopic medications, chronic medications, HR, MAP, syncope, cirrhosis, BP, melena, Hb, stroke, cardiovascular, renal, and respiratory diseases as input (116).

A long short-term memory (LSTM) Network by Shung et al. (117) indicates erythrocyte (RBC) transfusion for acute GIB (AGIB) cases. This model uses age, sex, vital signs, and laboratory factors, including blood gas, WBC, Hb, HCT, RBC, thrombocytes, coagulation tests, serum electrolytes, Cr, BUN, glucose, liver function tests, blood tests for iron, muscle function, lactate test, thyroid-stimulating hormone (TSH), vancomycin, urine Cr, sodium, and specific gravity (SG). The population was 4,050, and AUROC is 0.81. This model has a better performance in comparison with discrete time-regression models in the prediction of the need for packed RBC transfusion and can lead to less ischemic end-organ damage in AGIB patients (117).

### 5.2 Infant UGIB model

The BLOVO infant score by Quitadamo et al. (118) is a clinical assessment tool to forecast the UGI endoscopy necessity in infants who manifest hematemesis. This evidence-driven scoring system integrates bleeding historical data, clinical indicators, and laboratory results to evaluate the urgency of conducting endoscopy in infants exhibiting hematemesis. The primary objective of the BLOVO infant score is to assist healthcare professionals in making well-informed judgments concerning the timing of endoscopic assessment, thereby facilitating timely and suitable intervention for infants experiencing GIB (118).

### 5.3 Acute variceal bleeding (AVB) models

AVB can be a serious complication of portal hypertension. As a result, scoring systems for liver failure evaluation can be used to assess the AVB prognosis. Two commonly used diagnostic scoring methods for liver disease are the Child-Turcotte-Pugh (CTP) and the Model for End-Stage Liver Disease (MELD) scores.

In 1973, Pugh introduced the Child-Turcotte-Pugh (CTP) score as an adaption of the Child-Turcotte classification. This scoring system was initially developed to evaluate outcomes in patients undergoing surgery for portal hypertension (119). Since 1989, it has also been used to predict the outcome of patients undergoing transjugular intrahepatic portosystemic shunt (TIPS) procedures (120, 121). The CTP scoring system comprises five parameters: ascites, hepatic encephalopathy, international normalized ratio (INR), total serum bilirubin, and serum albumin levels. Each parameter is rate on one of three risk levels, and the overall score is used to classify patients into stages “A (5, 6)”, “B (7–9)”, or “C (9–14)”. According to the CTP score, patients categorized as “A” are considered to be better operative candidates compared to those in “C” (119).

While CTP scoring system is valuable, some limitations promoted the development of MELD score. MELD provides a more objective measure of liver disease severity using a mathematical formula based on serum bilirubin, serum creatinine, and INR levels. This model was claimed to offer a more precise assessment of liver function and a better prediction of patient survival, particularly for those awaiting liver transplants (121, 122).

There are controversies regarding effectiveness of MELD and CTP scores in predicting risk of rebleeding and mortality in AVBs. Regarding rebleeding, Aluizio et al. found no clinical value (area under the ROC curve <0.7) for Rockall, Blatchford, AIMS65, CTP, and MELD scores in predicting 6-week rebleeding outcomes in 222 AVB patients (123). In alignment with this, a systematic review carried out in 2021 confirmed that none of these scoring systems effectively predict in-hospital rebleeding or follow-up rebleeding within 3 months after the initial bleeding event (124). However, CTP and Clinical Rockall score (CRS) were clinically effective (AUROCs 0.72) in predicting in-hospital rebleeding by investigating 330 cirrhotic patients with AVB (125).

Concerning mortality, a retrospective study involving 222 AVB patients demonstrated that CTP and MELD (AUROC of 0.74 and 0.72, respectively) outperformed Rockall, Blatchford, and AIMS65 (AUROC <0.7) in 6-week mortality prediction (123). Similar findings were observed for predicting in-hospital survival in a study of 217 portal hypertensive patients with upper gastrointestinal bleeding, where CTP (AUC of 0.9) and MELD (AUC of 0.8) performed better than GBS (AUC of 0.64) (126). Furthermore, a systematic review by investigating 28 articles came to conclusion that CTP score was the most reliable predictor for in-hospital mortality in AVB patients, followed by AIMS65 and MELD with pooled AUC of 0.82, 0.79, and 0.78, respectively. However, in long term (3-months follow-up mortality), MELD was the strongest predictors, followed by AIMS65, CTP, and Clinical Rockall with pooled AUC of 0.79, 0.77, 0.74, and 0.7, respectively (124). This was consistent with Tantai study involving 330 cirrhotic patients with AVB, which showed higher AUROCs for CTP (0.87) in predicting in-hospital mortality compared to 0.84 for MELD, while the difference was not significant. Contrary to this, based on Mandal et al. study on 75 cirrhotic patients with variceal bleeding, MELD (AUROC of 0.85) outperformed CTP (AUROC of 0.74) for predicting mortality (127). This performance of MELD was confirmed by another study on 198 cirrhotic patients with upper gastrointestinal bleeding who did not undergo endoscopy, which showed the superior accuracy of MELD compared to CTP score in assessing mortality risk with AUC of 0.81 vs. 0.7 (7).

Despite ongoing debate regarding predicting performance of traditional scores such as MELD and CTP for mortality and rebleeding in UGIB cases, development of alternative scoring systems can provide more accurate assessment. One such advancement is the platelet-albumin-bilirubin (PALBI) score, which has been detailed previously. Derived from a specific formula incorporating serum bilirubin, serum albumin, and platelet counts, it has been shown that PALBI outperformed MELD, ALBI, and CTP in both survival and in-hospital rebleeding prediction (within 1 week) in 1,517 cirrhotic patients with AVB. In this study, only PALBI and ALBI showed clinical value for predicting survival with AUROCs of >0.8, while for rebleeding MELD and ALBI had prognostic value as well, with AUROCs of 0.74 and 0.76 which was lower than that of PALBI (0.79) (128). In addition, Huy's study confirmed that only ALBI and PALBI provide prognostic value for early rebleeding prognosis (AUC > 0.7), while all Child-Pugh, MELD, PALBI, and ALBI had good value for in-hospital mortality prediction with no significant differences (AUC > 0.7) (129).

Another scoring system called Hospital Outcome Prediction following Endoscopic Variceal Ligation (HOPE-EVL) was developed by observing 980 patients with esophageal variceal bleeding treated with EVL (130). This model contains five parameters of systolic blood pressure, GSC score, total bilirubin level, creatinine level, and albumin level. While this score is just developed by information from one country and only AVB patients after EVL, it outperformed MELD (AUC: 0.85) and CTP (AUC: 0.8) with AUC of approximately 0.9 for predicting in-hospital morality, which can be considered for clinical decisions after hemostasis.

### 5.4 Model for both UGIB and LGIB

The age, blood tests, and comorbidities (ABC) score by Laursen et al. (82) is a prognostic tool for GIB mortality, both UGIB and LGIB. The population of UGIB was 4,019 and LGIB was 2,336 cases, and AUROC is 0.81 for UGIB and 0.84 for LGIB. This scoring system emerged in response to the limitations of existing scores in accurately forecasting outcomes for patients with GIB. The ABC score incorporates three main factors: the patient's age, results from specific blood tests, and the presence of any comorbidities (82).

### 5.5 Endoscopy-based model

Cedars-Sinai Medical Center Predictive Index (CSMCPI) by Hay et al. (131) is an endoscopy-based system that identifies in-hospital lethal outcomes of acute UGIB (AUGIB). CSMCPI includes endoscopic findings, time between bleeding starts and administration in ED, and patient hemodynamics. During the endoscopy, we look for PUD or erosive disease with or without signs of recent hemorrhage (SRH), Mallory Weiss tear, angiodysplasia, persistent UGI hemorrhage, varices, and UGI cancer (131).

Table 1 shows strengths and limitations of novel scores.

## 6 Conclusion

In conclusion, emergency departments around the world continue to face a significant issue when it comes to managing GIB, whether it be upper or lower. Effective risk classification methods are desperately needed to prioritize patient care, as there is a chance of fatalities, rebleeding, and blood transfusions, among other potential negative effects. Although many scoring systems have been created to evaluate the severity and forecast results of GIB, it is still unclear which one is best for every clinical situation, but GBS remains the most widely used scoring system despite some limitations. By offering an extensive overview and comparison of several risk stratification score systems, this study seeks to close this gap. Our goal is to assist physicians in making well-informed decisions regarding the best care for patients with GIB by assessing the advantages and disadvantages of various grading systems.

## 7 Future perspective

With the increasing complexity of clinical presentations and the rising demand for precision medicine, future efforts should focus on dynamic and adaptable risk prediction models. Artificial intelligence-driven tools, capable of integrating real-time data from electronic health records, have the potential to outperform static scoring systems by providing more individualized and context-sensitive predictions.

Moreover, the development of hybrid models—combining traditional clinical variables with continuous physiologic monitoring—may enhance early warning capabilities, especially in settings with limited endoscopic access.

Future research should aim for large-scale prospective validation of these tools, and interdisciplinary collaboration is essential to ensure their safe and ethical integration into clinical practice.

## References

[1] DiGregorio AM Alvey H. Gastrointestinal Bleeding. (2019). 30725976

[2] Kim JJ Sheibani S Park S Buxbaum J Laine L. Causes of bleeding and outcomes in patients hospitalized with upper gastrointestinal bleeding. J Clin Gastroenterol. (2014) 48:113–8. 10.1097/MCG.0b013e318297fb40 23685847

[3] Marks I Janmohamed IK Malas S Mavrou A Banister T Patel N. Derivation and validation of a novel risk score to predict need for haemostatic intervention in acute upper gastrointestinal bleeding (London Haemostat Score). BMJ Open Gastroenterol. (2023) 10:e001008. 10.1136/bmjgast-2022-001008 36997237 PMC10069503

[4] Saade MC Kerbage A Jabak S Makki M Barada K Shaib Y. Validation of the new ABC score for predicting 30-day mortality in gastrointestinal bleeding. BMC Gastroenterol. (2022) 22:301. 10.1186/s12876-022-02374-y 35729498 PMC9209314

[5] Almaghrabi M Gandhi M Guizzetti L Iansavichene A Yan B Wilson A. Comparison of risk scores for lower gastrointestinal bleeding: a systematic review and meta-analysis. JAMA Netw Open. (2022) 5:e2214253. 10.1001/jamanetworkopen.2022.14253 35622365 PMC9142877

[6] Allo G Bürger M Gillessen J Kasper P Franklin J Mück V. Comparison of pre-endoscopic C-WATCH score with established risk assessment tools in patients with upper gastrointestinal bleeding. Dig Dis. (2022) 40:826–34. 10.1159/000522121 35073555 PMC9808639

[7] Cazacu SM Alexandru DO Statie RC Iordache S Ungureanu BS Iovanescu VF. The accuracy of pre-endoscopic scores for mortality prediction in patients with upper GI bleeding and no endoscopy performed. Diagnostics. (2023) 13:1188. 10.3390/diagnostics13061188 36980496 PMC10047350

[8] Franco MC Jang S Martins BDC Stevens T Jairath V Lopez R. Risk stratification in cancer patients with acute upper gastrointestinal bleeding: comparison of Glasgow-Blatchford, Rockall and AIMS65, and development of a new scoring system. Clin Endosc. (2022) 55:240–7. 10.5946/ce.2021.115 35052025 PMC8995992

[9] Schembre DB Ely RE Connolly JM Padhya KT Sharda R Brandabur JJ. Semiautomated Glasgow-Blatchford Bleeding Score helps direct bed placement for patients with upper gastrointestinal bleeding. BMJ Open Gastroenterol. (2020) 7:e000479. 10.1136/bmjgast-2020-000479 33214231 PMC7681917

[10] Blatchford O Murray WR Blatchford MJTL. A risk score to predict need for treatment for uppergastrointestinal haemorrhage. Lancet. (2000) 356:1318–21. 10.1016/S0140-6736(00)02816-6 11073021

[11] Stanley A Ashley D Dalton H Mowat C Gaya D Thompson E. Outpatient management of patients with low-risk upper-gastrointestinal haemorrhage: multicentre validation and prospective evaluation. Lancet. (2009) 373:42–7. 10.1016/S0140-6736(08)61769-9 19091393

[12] Barkun AN Bardou M Kuipers EJ Sung J Hunt RH Martel M. International consensus recommendations on the management of patients with nonvariceal upper gastrointestinal bleeding. Ann Intern Med. (2010) 152:101–13. 10.7326/0003-4819-152-2-201001190-00009 20083829

[13] Lim LG Ho KY Chan YH Teoh PL Khor CJ Lim LL. Urgent endoscopy is associated with lower mortality in high-risk but not low-risk nonvariceal upper gastrointestinal bleeding. Endoscopy. (2011) 43:300–6. 10.1055/s-0030-1256110 21360421

[14] Bryant RV Kuo P Williamson K Yam C Schoeman MN Holloway RH. Performance of the Glasgow-Blatchford score in predicting clinical outcomes and intervention in hospitalized patients with upper GI bleeding. Gastrointest Endosc. (2013) 78:576–83. 10.1016/j.gie.2013.05.003 23790755

[15] Laursen SB Dalton HR Murray IA Michell N Johnston MR Schultz M. Performance of new thresholds of the Glasgow Blatchford score in managing patients with upper gastrointestinal bleeding. Clin Gastroenterol Hepatol. (2015) 13:115–21.e2. 10.1016/j.cgh.2014.07.023 25058843

[16] In KR Oh YE Moon HS Jung S Kang SH Sung JK. Comparison and validation of several scoring systems for non-variceal upper gastrointestinal bleeding: a retrospective study. Sci Rep. (2024) 14:27940. 10.1038/s41598-024-79643-1 39537867 PMC11561243

[17] Cheng D Lu Y Teller T Sekhon H. Wu B. A modified Glasgow Blatchford Score improves risk stratification in upper gastrointestinal bleed: a prospective comparison of scoring systems. Aliment Pharmacol Ther. (2012) 36:782–9. 10.1111/apt.12029 22928529

[18] Rivieri S Carron P-N Schoepfer A Ageron F-X. External validation and comparison of the Glasgow-Blatchford score, modified Glasgow-Blatchford score, Rockall score and AIMS65 score in patients with upper gastrointestinal bleeding: a cross-sectional observational study in Western Switzerland. Eur J Emerg Med. (2023) 30:32–9. 10.1097/MEJ.0000000000000983 36542335 PMC10405788

[19] Quach DT Dao NH Dinh MC Nguyen CH Ho LX Nguyen N-DT. The performance of a modified Glasgow Blatchford score in predicting clinical interventions in patients with acute nonvariceal upper gastrointestinal bleeding: a Vietnamese prospective multicenter cohort study. Gut Liver. (2016) 10:375. 10.5009/gnl15254 26601829 PMC4849690

[20] Ko I-G Kim S-E Chang BS Kwak MS Yoon JY Cha JM. Evaluation of scoring systems without endoscopic findings for predicting outcomes in patients with upper gastrointestinal bleeding. BMC Gastroenterol. (2017) 17:1–8. 10.1186/s12876-017-0716-4 29233096 PMC5727876

[21] Pillai JB Vegas A Brister S. Thoracic complications of nasogastric tube: review of safe practice. Interact Cardiovasc Thorac Surg. (2005) 4:429–33. 10.1510/icvts.2005.109488 17670450

[22] Saleem M Kinshuck A Swift AJB. Nasogastric Tubes. (2010). p. 341.

[23] Kim SS Kim KU Kim SJ Seo SI Kim HS Jang MK. Predictors for the need for endoscopic therapy in patients with presumed acute upper gastrointestinal bleeding. Korean J Intern Med. (2019) 34:288. 10.3904/kjim.2016.406 29232942 PMC6406092

[24] Iwasaki H Shimura T Yamada T Aoki M Nomura S Kusakabe A. Novel nasogastric tube-related criteria for urgent endoscopy in nonvariceal upper gastrointestinal bleeding. Dig Dis Sci. (2013) 58:2564–71. 10.1007/s10620-013-2706-x 23695871

[25] Huang ES Karsan S Kanwal F Singh I Makhani M Spiegel B. Impact of nasogastric lavage on outcomes in acute GI bleeding. Gastrointest Endosc. (2011) 74:971–80. 10.1016/j.gie.2011.04.045 21737077

[26] Gilbert DA Silverstein FE Tedesco FJ Buenger NK Persing J. The national ASGE survey on upper gastrointestinal bleeding. III Endoscopy in upper gastrointestinal bleeding. Gastrointest Endosc. (1981) 27:94–102. 10.1016/S0016-5107(81)73157-2 6971777

[27] Silverstein FE Gilbert DA Tedesco FJ Buenger NK Persing J. The national ASGE survey on upper gastrointestinal bleeding. II Clinical prognostic factors. Gastrointest Endosc. (1981) 27:80–93. 10.1016/S0016-5107(81)73156-0 6971776

[28] Perng CL Lin HJ Chen CJ Lee FY Lee SD Lee CH. Characteristics of patients with bleeding peptic ulcer requiring emergency endoscopy and aggressive treatment. Am J Gastroenterol. (1994) 89:1811–4. 7942673

[29] Aljebreen AM Fallone CA Barkun AN. Nasogastric aspirate predicts high-risk endoscopic lesions in patients with acute upper-GI bleeding. Gastrointest Endosc. (2004) 59:172–8. 10.1016/S0016-5107(03)02543-4 14745388

[30] Wakatsuki T Mannami T Furutachi S Numoto H Umekawa T Mitsumune M. Glasgow-Blatchford score combined with nasogastric aspirate as a new diagnostic algorithm for patients with nonvariceal upper gastrointestinal bleeding. DEN Open. (2023) 3:e185. 10.1002/deo2.185 36397985 PMC9663679

[31] Gong EJ Hsing LC Seo HI Seo M Jun BG Park JK. Selected nasogastric lavage in patients with nonvariceal upper gastrointestinal bleeding. BMC Gastroenterol. (2021) 21:113. 10.1186/s12876-021-01690-z 33676407 PMC7937281

[32] Rockall TA Logan RF Devlin HB Northfield TC. Risk assessment after acute upper gastrointestinal haemorrhage. Gut. (1996) 38:316–21. 10.1136/gut.38.3.316 8675081 PMC1383057

[33] Vreeburg E Terwee C Snel P Rauws E Bartelsman J Vd Meulen J. Validation of the Rockall risk scoring system in upper gastrointestinal bleeding. Gut. (1999) 44:331–5. 10.1136/gut.44.3.331 10026316 PMC1727413

[34] Bessa X O'Callaghan E Ballesté B Nieto M Seoane A Panadès A. Applicability of the Rockall score in patients undergoing endoscopic therapy for upper gastrointestinal bleeding. Dig Liver Dis. (2006) 38:12–7. 10.1016/j.dld.2005.05.012 16314150

[35] Soncini M Triossi O Leo P Magni G Bertelè AM Grasso T. Management of patients with nonvariceal upper gastrointestinal hemorrhage before and after the adoption of the Rockall score, in the Italian Gastroenterology Units. Eur J Gastroenterol Hepatol. (2007) 19:543–7. 10.1097/MEG.0b013e3281532b89 17556899

[36] Rockall T Logan R Devlin H Northfield T. Influencing the practice and outcome in acute upper gastrointestinal haemorrhage. Gut. (1997) 41:606–11. 10.1136/gut.41.5.606 9414965 PMC1891577

[37] Enns RA Gagnon YM Barkun AN Armstrong D Gregor JC Fedorak RN. Validation of the Rockall scoring system for outcomes from non-variceal upper gastrointestinal bleeding in a Canadian setting. World J Gastroenterol. (2006) 12:7779. 10.3748/wjg.v12.i48.7779 17203520 PMC4087542

[38] Frías-Ordoñez JS Arjona-Granados DA Urrego-Díaz JA Briceño-Torres M Martínez-Marín JD. Validation of the rockall score in upper gastrointestinal tract bleeding in a colombian tertiary hospital. Arq Gastroenterol. (2022) 59:80–8. 10.1590/s0004-2803.202200001-15 35442342

[39] García Encinas C Paredes EB Guzmán Rojas P Gallegos López R Corzo Maldonado M Aguilar Sánchez V. Validación del score de Rockall en pacientes adultos mayores con hemorragia digestiva alta no variceal en un hospital general de tercer nivel. Revista de Gastroenterologí*a del Perú*. (2015) 35:25–31. 25875515

[40] Phang TS Vornik V Stubbs R. Risk assessment in upper gastrointestinal haemorrhage: implications for resource utilisation. New Zeal Med J. (2000) 113:331. 11008609

[41] Wang C-Y Qin J Wang J Sun C-Y Cao T Zhu D-D. Rockall score in predicting outcomes of elderly patients with acute upper gastrointestinal bleeding. World J Gastroenterol. (2013) 19:3466. 10.3748/wjg.v19.i22.3466 23801840 PMC3683686

[42] Johnston MR Murray IA Schultz M McLeod P O'Donnell N Norton H. Does preendoscopy rockall score safely identify low risk patients following upper gastrointestinal haemorrhage? Gastroenterol Res Pract. (2015) 2015:410702. 10.1155/2015/410702 26089867 PMC4451575

[43] García Encinas C Bravo Paredes E Guzmán Rojas P Gallegos López R Corzo Maldonado M Aguilar Sánchez V. Validation of the Rockall score in elderly patients with non variceal upper gastrointestinal bleeding in a third level general hospital. Rev Gastroenterol Peru. (2015) 35:25–31. 25875515

[44] Lip HT Heah HT Huei TJ Premaa S Sarojah A. Rockall risk score in predicting 30 days non-variceal upper gastrointestinal rebleeding in a Malaysian population. Med J Malaysia. (2016) 71:225–30. 28064286

[45] Lu M Sun G Huang H Zhang X Xu Y Chen S. Comparison of the Glasgow-Blatchford and Rockall Scores for prediction of nonvariceal upper gastrointestinal bleeding outcomes in Chinese patients. Medicine. (2019) 98:e15716. 10.1097/MD.0000000000015716 31124950 PMC6571241

[46] Taslidere B Sonmez E Özcan AB Mehmetaj L Keskin EB Gulen B. Comparison of the quick SOFA score with Glasgow-Blatchford and Rockall scores in predicting severity in patients with upper gastrointestinal bleeding. Am J Emerg Med. (2021) 45:29–36. 10.1016/j.ajem.2021.02.016 33647759

[47] Yang EH Cheng HC Wu CT Chen WY Lin MY Sheu BS. Peptic ulcer bleeding patients with Rockall scores ≥6 are at risk of long-term ulcer rebleeding: A 3 5-year prospective longitudinal study. J Gastroenterol Hepatol. (2018) 33:156–63. 10.1111/jgh.13822 28497645

[48] Bozkurt MA Peker KD Unsal MG Yirgin H Kahraman I Aliş H. The importance of Rockall scoring system for upper gastrointestinal bleeding in long-term follow-up. Indian J Surg. (2017) 79:188–91. 10.1007/s12262-015-1434-1 28659669 PMC5473785

[49] Custovic N Husic-Selimovic A Srsen N Prohic D. Comparison of Glasgow-Blatchford score and Rockall score in patients with upper gastrointestinal bleeding. Medical Arch. (2020) 74:270–4. 10.5455/medarh.2020.74.270-274 33041443 PMC7520069

[50] Chandra S Hess EP Agarwal D Nestler DM Montori VM Song LM. External validation of the Glasgow-Blatchford Bleeding Score and the Rockall Score in the US setting. Am J Emerg Med. (2012) 30:673–9. 10.1016/j.ajem.2011.03.010 21641145

[51] Morarasu BC Sorodoc V Haisan A Morarasu S Bologa C Haliga RE. Age, blood tests and comorbidities and AIMS65 risk scores outperform Glasgow-Blatchford and pre-endoscopic Rockall score in patients with upper gastrointestinal bleeding. World J Clini Cases. (2023) 11:4513–30. 10.12998/wjcc.v11.i19.4513 37469720 PMC10353516

[52] Saltzman JR Tabak YP Hyett BH Sun X Travis AC Johannes RS. simple risk score accurately predicts in-hospital mortality, length of stay, and cost in acute upper GI bleeding. Gastrointest Endosc. (2011) 74:1215–24. 10.1016/j.gie.2011.06.024 21907980

[53] Hirai R Shimodate Y Minami M Ishikawa S Kanadani T Takezawa R. AIMS65 +6++predicts prognosis of patients with duodenal ulcer bleeding; a comparison with other risk-scoring systems. Eur J Gastroenterol Hepatol. (2021) 33:1480–4. 10.1097/MEG.0000000000002010 33252414

[54] Robertson M Ng J Abu Shawish W Swaine A Skardoon G Huynh A. Risk stratification in acute variceal bleeding: comparison of the AIMS65 score to established upper gastrointestinal bleeding and liver disease severity risk stratification scoring systems in predicting mortality and rebleeding. Dig Endosc. (2020) 32:761–8. 10.1111/den.13577 31863515

[55] Chang A Ouejiaraphant C Akarapatima K Rattanasupa A Prachayakul V. Prospective comparison of the AIMS65 Score, Glasgow-Blatchford Score, and Rockall Score for predicting clinical outcomes in patients with variceal and nonvariceal upper gastrointestinal bleeding. Clin Endosc. (2021) 54:211–21. 10.5946/ce.2020.068 32668528 PMC8039743

[56] Lu X Zhang X Chen H. Comparison of the AIMS65 score with the Glasgow-Blatchford and Rockall scoring systems for the prediction of the risk of in-hospital death among patients with upper gastrointestinal bleeding. Revista espanola de enfermedades digestivas. (2020) 112:467–73. 10.17235/reed.2020.6496/2019 32379473

[57] Akhila Arya PV Thulaseedharan NK Raj R Unnikrishnan DC Jacob A. AIMS65, Glasgow-Blatchford bleeding score and modified Glasgow-Blatchford bleeding score in predicting outcomes of upper gastrointestinal bleeding: An accuracy and calibration study. Indian J Gastroenterol. (2023) 42:496–504. 10.1007/s12664-023-01387-z 37382854

[58] Lee MW Lee HJ Shin KH Kim GH Kim HH. Red blood cell transfusion volumes according to AIMS65 scores in patients with peptic ulcer bleeding. Lab Med. (2022) 53:190–3. 10.1093/labmed/lmab080 34522953

[59] Cúrdia Gonçalves T Barbosa M Xavier S Boal Carvalho P Magalhães J Marinho C. AIMS65 score: a new prognostic tool to predict mortality in variceal bleeding. Scand J Gastroenterol. (2017) 52:469–70. 10.1080/00365521.2016.1260155 27887038

[60] Marmo R Koch M Cipolletta L Capurso L Grossi E Cestari R. Predicting mortality in non-variceal upper gastrointestinal bleeders: validation of the Italian PNED Score and Prospective Comparison with the Rockall Score. Am J Gastroenterol. (2010) 105:1284–91. 10.1038/ajg.2009.687 20051943

[61] Matsuhashi T Hatta W Hikichi T Fukuda S Mikami T Tatsuta T. A simple prediction score for in-hospital mortality in patients with nonvariceal upper gastrointestinal bleeding. J Gastroenterol. (2021) 56:758–68. 10.1007/s00535-021-01797-w 34143312

[62] Hoffmann V Neubauer H Heinzler J Smarczyk A Hellmich M Bowe A. A novel easy-to-use prediction scheme for upper gastrointestinal bleeding: cologne-WATCH (C-WATCH) risk score. Medicine. (2015) 94:e1614. 10.1097/MD.0000000000001614 26402828 PMC4635768

[63] Oakland K Kahan BC Guizzetti L Martel M Bryant RV Brahmania M. Development, validation, and comparative assessment of an international scoring system to determine risk of upper gastrointestinal bleeding. Clin Gastroenterol Hepatol. (2019) 17:1121–9.e2. 10.1016/j.cgh.2018.09.039 30268566

[64] Tammaro L Buda A Di Paolo MC Zullo A Hassan C Riccio E. A simplified clinical risk score predicts the need for early endoscopy in non-variceal upper gastrointestinal bleeding. Dig Liver Dis. (2014) 46:783–7. 24953205 10.1016/j.dld.2014.05.006

[65] Sasaki Y Abe T Kawamura N Keitoku T Shibata I Ohno S. Prediction of the need for emergency endoscopic treatment for upper gastrointestinal bleeding and new score model: a retrospective study. BMC Gastroenterol. (2022) 22:337. 10.1186/s12876-022-02413-8 35820868 PMC9277905

[66] Redondo-Cerezo E Vadillo-Calles F Stanley AJ Laursen S Laine L Dalton HR. MAP(ASH): A new scoring system for the prediction of intervention and mortality in upper gastrointestinal bleeding. J Gastroenterol Hepatol. (2020) 35:82–9. 10.1111/jgh.14811 31359521

[67] Marmo R Soncini M Bucci C Occhipinti V Pellegrini L Zullo A. Derivation and validation of Re.Co.De death score risk in patients with acute nonvariceal upper GI bleeding. Gastrointest Endosc. (2022) 96:36–43.e8. 35150665 10.1016/j.gie.2022.01.024

[68] Iino C Mikami T Igarashi T Aihara T Ishii K Sakamoto J. Evaluation of scoring models for identifying the need for therapeutic intervention of upper gastrointestinal bleeding: a new prediction score model for Japanese patients. Dig Endosc. (2016) 28:714–21. 10.1111/den.12666 27061908

[69] Saeed ZA Ramirez FC Hepps KS Cole RA Graham DY. Prospective validation of the Baylor bleeding score for predicting the likelihood of rebleeding after endoscopic hemostasis of peptic ulcers. Gastrointest Endosc. (1995) 41:561–5. 10.1016/S0016-5107(95)70191-5 7672549

[70] Ito N Funasaka K Fujiyoshi T Furukawa K Kakushima N Furune S. Modified N score is helpful for identifying patients who need endoscopic intervention among those with black stools without hematemesis. Dig Endosc. (2022) 34:1157–65. 10.1111/den.14323 35396885

[71] Gralnek IM Neeman Z Strate LL. Acute lower gastrointestinal bleeding. N Engl J Med. (2017) 376:1054–63. 10.1056/NEJMcp1603455 28296600

[72] Strate LL Gralnek IM ACG. Clinical guideline: management of patients with acute lower gastrointestinal bleeding. Am J Gastroenterol. (2016) 111:459–74. 10.1038/ajg.2016.41 26925883 PMC5099081

[73] Niikura R Yasunaga H Yamaji Y Horiguchi H Fushimi K Yamada A. Factors affecting in-hospital mortality in patients with lower gastrointestinal tract bleeding: a retrospective study using a national database in Japan. J Gastroenterol. (2015) 50:533–40. 10.1007/s00535-014-0994-3 25181990

[74] Oakland K Jairath V Uberoi R Guy R Ayaru L Mortensen N. Derivation and validation of a novel risk score for safe discharge after acute lower gastrointestinal bleeding: a modelling study. Lancet Gastroenterol Hepatol. (2017) 2:635–43. 10.1016/S2468-1253(17)30150-4 28651935

[75] DiLena DD Bouvet SC Somers MJ Merchant MA Levin TR Rauchwerger AS. Oakland score to identify low-risk patients with lower gastrointestinal bleeding performs well among emergency department patients. Int J Emerg Med. (2025) 18:19. 10.1186/s12245-025-00815-5 39901084 PMC11792187

[76] Oakland K Kothiwale S Forehand T Jackson E Bucknall C Sey MSL. External validation of the oakland score to assess safe hospital discharge among adult patients with acute lower gastrointestinal bleeding in the US. JAMA Netw Open. (2020) 3:e209630. 10.1001/jamanetworkopen.2020.9630 32633766 PMC7341175

[77] Raqi I Potier P Lagasse JP. External validation of the Oakland Score for acute lower gastrointestinal bleeding. Cureus. (2024) 16:e57264. 10.7759/cureus.57264 38686245 PMC11056811

[78] Nieto LM Bezabih Y Narvaez SI Rouse C Perry C Vega KJ. Single center assessment of the role of Oakland score among patients admitted for acute lower gastrointestinal bleeding. BMC Gastroenterol. (2024) 24:225. 10.1186/s12876-024-03283-y 39009983 PMC11247859

[79] Prasitvarakul K Attanath N Chang A. Comparison of scoring systems for predicting clinical outcomes of acute lower gastrointestinal bleeding: a prospective cohort study. World J Surg. (2024) 48:474–83. 10.1002/wjs.12053 38686770

[80] Garcia-Iglesias P Machlab S Martinez-Bauer E Lira A Campo R Marín S. Diagnostic accuracy of the Oakland score vs. haemoglobin for predicting outcomes in lower gastrointestinal bleeding. Gastroenterol Hepatol. (2024) 47:742–9. 10.1016/j.gastrohep.2024.02.002 38341089

[81] Quach DT Vo UP Nguyen NT Le LT Vo MH Ho PT. An external validation study of the Oakland and Glasgow-Blatchford scores for predicting adverse outcomes of acute lower gastrointestinal bleeding in an Asian population. Gastroenterol Res Pract. (2021) 2021:8674367. 10.1155/2021/8674367 33505461 PMC7806364

[82] Laursen SB Oakland K Laine L Bieber V Marmo R Redondo-Cerezo E. ABC score: a new risk score that accurately predicts mortality in acute upper and lower gastrointestinal bleeding: an international multicentre study. Gut. (2021) 70:707–16. 10.1136/gutjnl-2019-320002 32723845

[83] Yeon SH Moon HS Choi SW Kang SH Sung JK Jeong HY. comparative study of scoring systems that accurately predict the prognosis of lower gastrointestinal bleeding. Int J Colorectal Dis. (2023) 38:51. 10.1007/s00384-023-04348-2 36806639

[84] Hreinsson JP Sigurdardottir R Lund SH Bjornsson ES. The SHA(2)PE score: a new score for lower gastrointestinal bleeding that predicts low-risk of hospital-based intervention. Scand J Gastroenterol. (2018) 53:1484–9. 10.1080/00365521.2018.1532019 30457020

[85] Cerruti T Maillard MH Hugli O. Acute lower gastrointestinal bleeding in an emergency department and performance of the SHA(2)PE score: a retrospective observational study. J Clin Med. (2021) 10:5476. 10.3390/jcm10235476 34884177 PMC8658478

[86] Gonzalez-Gonzalez L Iborra I Fortuny M Mañosa M Calm A Colan J. External validation of the SHA(2)PE score and its comparison to the Oakland score for the prediction of safe discharge in patients with lower gastrointestinal bleeding. Surg Endosc. (2024) 38:4468–75. 10.1007/s00464-024-10953-1 38902406

[87] Jeong KB Moon HS In KR Kang SH Sung JK Jeong HY. Which scoring systems are useful for predicting the prognosis of lower gastrointestinal bleeding? Old and new. BMC Gastroenterol. (2025) 25:49. 10.1186/s12876-025-03638-z 39891040 PMC11786468

[88] Aoki T Nagata N Shimbo T Niikura R Sakurai T Moriyasu S. Development and validation of a risk scoring system for severe acute lower gastrointestinal bleeding. Clin Gastroenterol Hepatol. (2016) 14:1562–70.e2. 10.1016/j.cgh.2016.05.042 27311620

[89] Aoki T Yamada A Nagata N Niikura R Hirata Y Koike K. External validation of the NOBLADS score, a risk scoring system for severe acute lower gastrointestinal bleeding. PLoS ONE. (2018) 13:e0196514. 10.1371/journal.pone.0196514 29698506 PMC5919702

[90] Brito M Patita M Nunes G Canhoto M Fonseca J. NOBLADS-External Validation of a Risk Scoring System for Severe Acute Lower Gastrointestinal Bleeding. Dis Colon Rectum. (2022) 65:264–70. 10.1097/DCR.0000000000002064 34636778

[91] Strate LL Orav EJ Syngal S. Early predictors of severity in acute lower intestinal tract bleeding. Arch Intern Med. (2003) 163:838–43. 10.1001/archinte.163.7.838 12695275

[92] Strate LL Saltzman JR Ookubo R Mutinga ML Syngal S. Validation of a clinical prediction rule for severe acute lower intestinal bleeding. Am J Gastroenterol. (2005) 100:1821–7. 10.1111/j.1572-0241.2005.41755.x 16086720

[93] Xavier SA Machado FJ Magalhães JT Cotter JB. Acute lower gastrointestinal bleeding: are STRATE and BLEED scores valid in clinical practice? Colorectal Dis. (2019) 21:357–64. 10.1111/codi.14529 30537416

[94] Tominaga N Sadashima E Aoki T Fujita M Kobayashi K Yamauchi A. A novel prediction tool for mortality in patients with acute lower gastrointestinal bleeding requiring emergency hospitalization: a large multicenter study. Sci Rep. (2024) 14:5367. 10.1038/s41598-024-55889-7 38438534 PMC10912311

[95] Kollef MH Canfield DA Zuckerman GR. Triage considerations for patients with acute gastrointestinal hemorrhage admitted to a medical intensive care unit. Crit Care Med. (1995) 23:1048–54. 10.1097/00003246-199506000-00009 7774215

[96] Kollef MH O'Brien JD Zuckerman GR Shannon W BLEED. a classification tool to predict outcomes in patients with acute upper and lower gastrointestinal hemorrhage. Crit Care Med. (1997) 25:1125–32. 10.1097/00003246-199707000-00011 9233736

[97] Fujita M Aoki T Manabe N Ito Y Kobayashi K Yamauchi A. LONG-HOSP score: a novel predictive score for length of hospital stay in acute lower gastrointestinal bleeding - a multicenter nationwide study. Digestion. (2023) 104:446–59. 10.1159/000531646 37536306 PMC10711765

[98] Sengupta N Tapper EB. Derivation and internal validation of a clinical prediction tool for 30-day mortality in lower gastrointestinal bleeding. Am J Med. (2017) 130:601.e1—e8. 10.1016/j.amjmed.2016.12.009 28065767

[99] Das A Ben-Menachem T Cooper GS Chak A Sivak MV Gonet JA. Prediction of outcome in acute lower-gastrointestinal haemorrhage based on an artificial neural network: internal and external validation of a predictive model. Lancet. (2003) 362:1261–6. 10.1016/S0140-6736(03)14568-0 14575969

[100] Newman J Fitzgerald JE Gupta S von Roon AC Sigurdsson HH Allen-Mersh TG. Outcome predictors in acute surgical admissions for lower gastrointestinal bleeding. Colorectal Dis. (2012) 14:1020–6. 10.1111/j.1463-1318.2011.02824.x 21910819

[101] Velayos FS Williamson A Sousa KH Lung E Bostrom A Weber EJ. Early predictors of severe lower gastrointestinal bleeding and adverse outcomes: a prospective study. Clin Gastroenterol Hepatol. (2004) 2:485–90. 10.1016/S1542-3565(04)00167-3 15181617

[102] Subbe CP Kruger M Rutherford P Gemmel L. Validation of a modified Early Warning Score in medical admissions. QJM. (2001) 94:521–6. 10.1093/qjmed/94.10.521 11588210

[103] Wu PH Hung SK Ko CA Chang CP Hsiao CT Chung JY. Performance of six clinical physiological scoring systems in predicting in-hospital mortality in elderly and very elderly patients with acute upper gastrointestinal bleeding in emergency department. Medicina. (2023) 59:556. 10.3390/medicina59030556 36984556 PMC10057917

[104] Lai YC Hung MS Chen YH Chen YC. Comparing AIMS65 Score with MEWS, qSOFA Score, Glasgow-Blatchford Score, and Rockall Score for predicting clinical outcomes in cirrhotic patients with upper gastrointestinal bleeding. J Acute Med. (2018) 8:154–67. 32995218 10.6705/j.jacme.201812_8(4).0003 PMC7517937

[105] Kim WY Lee J Lee JR Jung YK Kim HJ Huh JW. A risk scoring model based on vital signs and laboratory data predicting transfer to the intensive care unit of patients admitted to gastroenterology wards. J Crit Care. (2017) 40:213–7. 10.1016/j.jcrc.2017.04.024 28445859

[106] Bozkurt S Kose A Arslan ED Erdogan S Ucbilek E Cevik I. Validity of modified early warning, Glasgow Blatchford, and pre-endoscopic Rockall scores in predicting prognosis of patients presenting to emergency department with upper gastrointestinal bleeding. Scand J Trauma Resusc Emerg Med. (2015) 23:109. 10.1186/s13049-015-0194-z 26714636 PMC4696211

[107] Ho le O Li H Shahidah N Koh ZX Sultana P Hock Ong ME. Poor performance of the modified early warning score for predicting mortality in critically ill patients presenting to an emergency department. World J Emerg Med. (2013) 4:273–8. 10.5847/wjem.j.issn.1920-8642.2013.04.005 25215131 PMC4129901

[108] Vincent JL Moreno R Takala J Willatts S De Mendonça A Bruining H. The SOFA (Sepsis-related Organ Failure Assessment) score to describe organ dysfunction/failure. On behalf of the Working Group on Sepsis-Related Problems of the European Society of Intensive Care. Med Intens Care Med. (1996) 22:707–10. 10.1007/BF01709751 8844239

[109] Singer M Deutschman CS Seymour CW Shankar-Hari M Annane D Bauer M. The third international consensus definitions for sepsis and septic shock (Sepsis-3). JAMA. (2016) 315:801–10. 10.1001/jama.2016.0287 26903338 PMC4968574

[110] Knaus WA Draper EA Wagner DP Zimmerman JE APACHE II. a severity of disease classification system. Crit Care Med. (1985) 13:818–29. 10.1097/00003246-198510000-00009 3928249

[111] Charlson ME Pompei P Ales KL MacKenzie CR A. new method of classifying prognostic comorbidity in longitudinal studies: development and validation. J Chronic Dis. (1987) 40:373–83. 10.1016/0021-9681(87)90171-8 3558716

[112] Firat BT Gulen M Satar S Firat A Acehan S Isikber C. Perfusion index: Could this be a new triage tool for upper gastrointestinal system bleeding in the emergency department? A prospective cohort study. Sao Paulo Med J. (2021) 139:583–90. 10.1590/1516-3180.2021.0106.r1.0904221 34644767 PMC9634832

[113] Deshmukh F Merchant SS. Explainable machine learning model for predicting GI bleed mortality in the intensive care unit. Am J Gastroenterol. (2020) 115:1657–68. 10.14309/ajg.0000000000000632 32341266

[114] Herrin J Abraham NS Yao X Noseworthy PA Inselman J Shah ND. Comparative effectiveness of machine learning approaches for predicting gastrointestinal bleeds in patients receiving antithrombotic treatment. JAMA Netw Open. (2021) 4:e2110703. 10.1001/jamanetworkopen.2021.10703 34019087 PMC8140376

[115] Ungureanu BS Gheonea DI Florescu DN Iordache S Cazacu SM Iovanescu VF. Predicting mortality in patients with nonvariceal upper gastrointestinal bleeding using machine-learning. Front Med. (2023) 10:1134835. 10.3389/fmed.2023.1134835 36873879 PMC9982090

[116] Veisman I Oppenheim A Maman R Kofman N Edri I Dar L. A novel prediction tool for endoscopic intervention in patients with acute upper gastro-intestinal bleeding. J Clin Med. (2022) 11:5893. 10.3390/jcm11195893 36233760 PMC9573673

[117] Shung D Huang J Castro E Tay JK Simonov M Laine L. Neural network predicts need for red blood cell transfusion for patients with acute gastrointestinal bleeding admitted to the intensive care unit. Sci Rep. (2021) 11:8827. 10.1038/s41598-021-88226-3 33893364 PMC8065139

[118] Quitadamo P Anselmi F Mantegazza C Tambucci R Campanozzi A Malamisura M. Hematemesis in infants: the first evidence-based score to predict the need for timely endoscopy. Pediatr Emerg Care. (2022) 38:e1245–e50. 10.1097/PEC.0000000000002579 35482500

[119] Pugh RN Murray-Lyon IM Dawson JL Pietroni MC Williams R. Transection of the oesophagus for bleeding oesophageal varices. Br J Surg. (1973) 60:646–9. 10.1002/bjs.1800600817 4541913

[120] Richter GM Noeldge G Palmaz JC Roessle M. The transjugular intrahepatic portosystemic stent-shunt (TIPSS): results of a pilot study. Cardiovasc Intervent Radiol. (1990) 13:200–7. 10.1007/BF02575474 2121348

[121] Ruf A Dirchwolf M Freeman RB. From Child-Pugh to MELD score and beyond: Taking a walk down memory lane. Ann Hepatol. (2022) 27:100535. 10.1016/j.aohep.2021.100535 34560316

[122] Kamath PS Wiesner RH Malinchoc M Kremers W Therneau TM Kosberg CL. A model to predict survival in patients with end-stage liver disease. Hepatology. (2001) 33:464–70. 10.1053/jhep.2001.22172 11172350

[123] Aluizio CLS Montes CG Reis G Nagasako CK. Risk stratification in acute variceal bleeding: Far from an ideal score. Clinics. (2021) 76:e2921. 10.6061/clinics/2021/e2921 34190855 PMC8221560

[124] Yang L Sun R Wei N Chen H. Systematic review and meta-analysis of risk scores in prediction for the clinical outcomes in patients with acute variceal bleeding. Ann Med. (2021) 53:1806–15. 10.1080/07853890.2021.1990394 34661508 PMC8525940

[125] Tantai XX Liu N Yang LB Wei ZC Xiao CL Song YH. Prognostic value of risk scoring systems for cirrhotic patients with variceal bleeding. World J Gastroenterol. (2019) 25:6668–80. 10.3748/wjg.v25.i45.6668 31832005 PMC6906204

[126] Jamil Z Perveen S Khalid S Aljuaid M Shahzad M Ahmad B. Child-Pugh Score, MELD Score and Glasgow Blatchford Score to predict the in-hospital outcome of portal hypertensive patients presenting with upper gastrointestinal bleeding: an experience from tertiary healthcare system. J Clin Med. (2022) 11:6654. 10.3390/jcm11226654 36431131 PMC9693334

[127] Mandal AK Paudel MS Kc S Chaudhary S Paudel BN Poudyal NS. Factors predicting mortality of acute variceal bleeding in liver cirrhosis. JNMA J Nepal Med Assoc. (2018) 56:493–6. 10.31729/jnma.3408 30058631 PMC8997329

[128] Elshaarawy O Allam N Abdelsameea E Gomaa A Waked I. Platelet-albumin-bilirubin score - a predictor of outcome of acute variceal bleeding in patients with cirrhosis. World J Hepatol. (2020) 12:99–107. 10.4254/wjh.v12.i3.99 32231763 PMC7097503

[129] Huy DQ Chung NV Dong DT. Value of some scoring systems for the prognosis of rebleeding and in-hospital mortality in liver cirrhosis with acute variceal bleeding. Gastroenterol Insights. (2023) 14:144–55. 10.3390/gastroent14020011

[130] Ichita C Goto T Okada Y Uojima H Iwagami M Sasaki A. Development and validation of a scoring system for in-hospital mortality following band ligation in esophageal variceal bleeding. Dig Endosc. (2024) 36:1105–14. 10.1111/den.14773 38462957

[131] Hay JA Lyubashevsky E Elashoff J Maldonado L Weingarten SR Ellrodt AG. Upper gastrointestinal hemorrhage clinical–guideline determining the optimal hospital length of stay. Am J Med. (1996) 100:313–22. 10.1016/S0002-9343(97)89490-9 8629677

[132] Roayaie S Jibara G Berhane S Tabrizian P Park J-W Yang J. PALBI-an objective score based on platelets, albumin & bilirubin stratifies HCC patients undergoing resection & ablation better than Child's classification. In: Hepatology. Hoboken: Wiley-Blackwell. (2015).

